# LIPIODOL LYMPHANGIOGRAPHY AND EMBOLIZATION OF CHYLOUS ASCITES AFTER PANCREATODUODENECTOMY

**DOI:** 10.1590/0102-672020220002e1677

**Published:** 2022-09-09

**Authors:** Orlando Jorge Martins Torres, Natália Helena Valleta, José Maria Assunção Moraes-Junior, Milena Vasconcelos Falcão, Joana Marques Lobo Quariguasi, Igor Murad Faria

**Affiliations:** 1 Hospital São Domingos, Department of Gastrointestinal Surgery – São Luiz (MA), Brazil; 2 Universidade Federal do Maranhão, Department of Gastrointestinal and Transplant Surgery – São Luiz (MA), Brazil; 3 Hospital São Domingos, Department of Oncology – São Luiz (MA), Brazil; 4 Hospital São Domingos, Department of Interventional Radiology – São Luiz (MA), Brazil.

**Keywords:** Chylous Ascites, Lymphatic System, Ethiodol, Lymphography, Catheterization, Radiology, Interventional, Ascite Quilosa, Sistema Linfático, Óleo Etiodado, Linfografia, Cateterismo, Radiologia Intervencionista

## INTRODUCTION

Chylous ascites is a rare disorder characterized by the accumulation of triglyceride-rich fluid in the abdominal cavity due to the disruption of the lymphatic system^2,3,11^. A variety of conditions can cause chylous ascites, including congenital lymphatic abnormalities, inflammatory and infectious conditions, liver cirrhosis, malignancies, cardiogenic trauma, and iatrogenic injury postsurgery^2,8^. The fluid of chylous ascites has a “milky” appearance, and the triglyceride content is over 110 mg/dL^2,11^. Chylous ascites cause mechanical symptoms related to abdominal distension and may lead to malnutrition and impairment of the immune system that is associated with morbidity and mortality. The prognosis is different in chylous ascites and can be fatal depending on the underlying cause. High mortality rates have been observed in up to 90% of patients with cancer if not treated properly^1,4,11^.

There is no standardized treatment of chylous ascites, and the current management includes high-protein and low-fat diet as conservative treatment, total parenteral nutrition (TPN), paracentesis, radiology, and surgical interventions. Lymphangiography and percutaneous embolization are a less invasive procedure and have been reported to have a therapeutic effect in 56–86% of patients with lymphatic leaks^1,2^. Intranodal lymphatic embolization has been previously described in the treatment of some visceral lymphatic leaks^1,5,13^. This study aimed to present a case of chylous ascites after pancreatoduodenectomy treated with lipiodol lymphangiography and embolization.

## CASE REPORT

A 67-year-old male patient with previous chronic pancreatitis presented with jaundice and weight loss. Computed tomography scan revealed a solid mass in the pancreatic head. Magnetic resonance image and cholangiopancreatography showed a pancreatic (10.2 mm) and bile duct (12 mm) dilated, and solid mass and calcification in the pancreatic head. Liver metastasis, ascites, suspected lymph nodes, or signs of peritoneal disease were not observed. After preoperative evaluation, the patient underwent pancreatoduodenectomy with total mesopancreas excision and lymphadenectomy. The postoperative course was uneventful, the length of ICU stay was 3 days, and the patient was discharged on postoperative day 9. The pathologic study confirmed pancreatic ductal adenocarcinoma, and the patient was sent for adjuvant chemotherapy.

**Figure 1:**
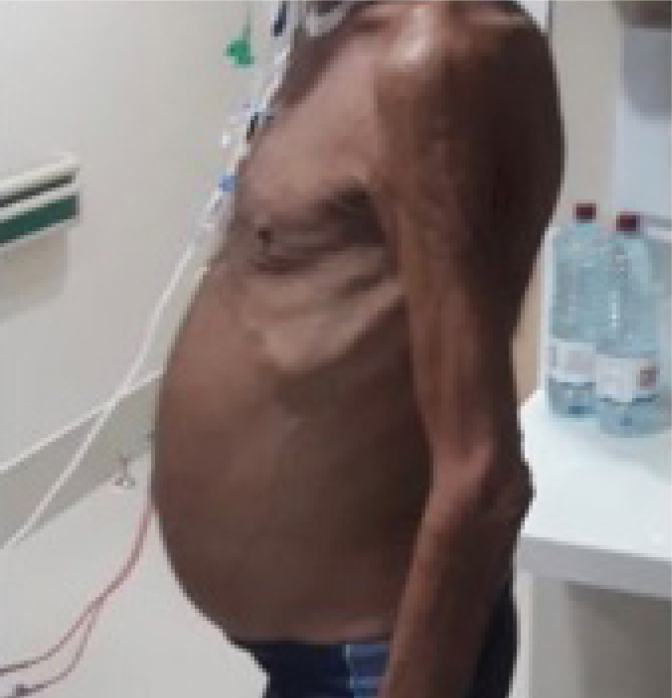
Patient presenting ascites after pancreatoduodenectomy.

On postoperative day 50, the patient presented to our institution with abdominal distension, weight loss, malnutrition, and bilateral lower extremity edema (Figure 1). Abdominal ultrasonography demonstrated large-volume ascites. The patient underwent paracentesis, 4.0 L of milky fluid was removed, confirming chylous ascites. Fluid amylase and lactate dehydrogenase were normal. Fluid cytology was negative for malignancy.

During the hospitalization, the patient was managed with conservative measures, including high-protein, low fat, medium-chain triglyceride diet and albumin replacement. Due to the rapid accumulation of ascites after paracentesis and no clinical improvement, we discussed the case with the interventional radiology team and decided for lipiodol lymphangiography followed by embolization.

**Figure 2:**
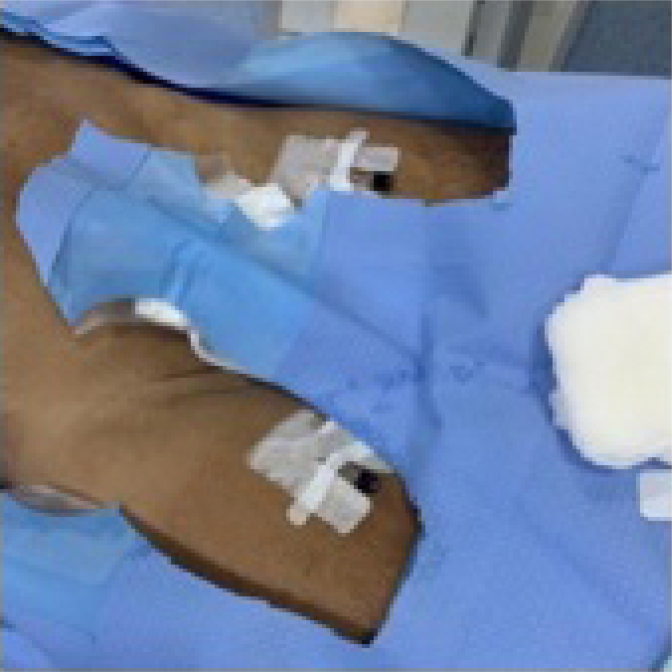
Bilateral inguinal lymph nodes access for lymphangiography

**Figure 3:**
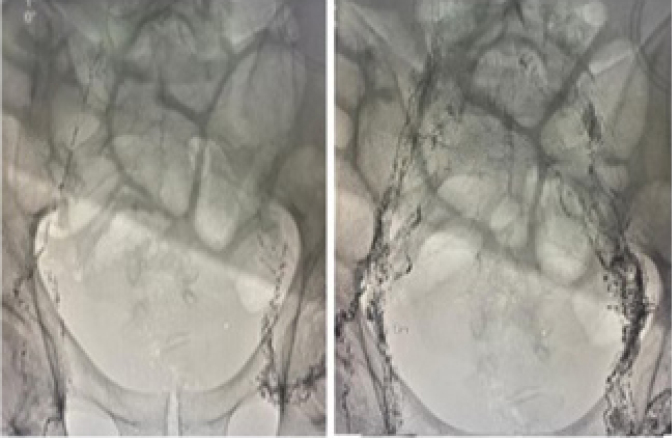
(A) Lipiodol lymphangiography (B) after lymph nodes puncture

**Figure 4:**
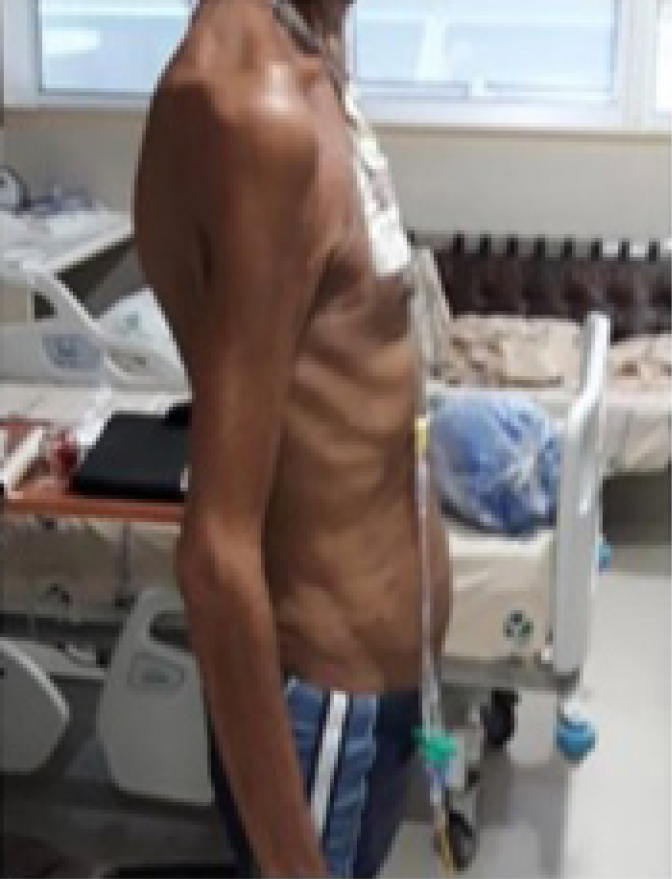
Patient 9 days after lymphangiography and embolization.

Bilateral inguinal lymph nodes were identified and accessed using ultrasound guidance (Figure 2). The needle position was confirmed near the lymphatic hila, lipiodol was slowly injected, and fluoroscopy images were then obtained of the retroperitoneal lymphatic channels. During the lymphangiography, no active extravasation was identified. However, embolization was performed as part of the routine treatment (Figure 3A and 3B). The procedure was performed uneventful, the atient remained stable, and he was discharged 9 days after the procedure, without chylous ascites (Figure 4). No additional paracenteses were necessary during the following 5-month follow-up period, and the patient is now under adjuvant chemotherapy. Written informed consent was obtained from the patient for publication of this report and accompanying images.

## DISCUSSION

Pancreatoduodenectomy is a technically challenging surgical procedure with the incidence of postoperative complications ranging from 30 to 61%. The more common complications include delayed gastric emptying, postoperative pancreatic fistula, postoperative bleeding, and infectious complications^14^. Delayed gastric emptying is a frequent complication after pancreatoduodenectomy and it is associated with prolonged hospital stay and high costs, even with the low mortality rate resulting from it^10^. Chylous ascites is a rare form of ascites resulting from the leakage of lipid-rich intestinal lymph into the peritoneal cavity and may be an iatrogenic postoperative complication^2,7,12^.

As far as we know, there is no report about chylous ascites following pancreatoduodenectomy and few reports of the management of chylous ascites by lymphangiography and embolization. Surgeons should be aware of the possibility of this complication. Although rare, chyle leak is a highly morbid condition and, when persistent, can lead to life-threatening complications such as malnutrition, weight loss, infection, immunodeficiency, and prolonged hospital stay^6,9,12^. Thus, early diagnosis and intervention is essential to minimize morbidity and mortality. Chylous leaks can happen anywhere along the pathway of chyle that begins in the intestinal lymphatic ducts and continue through the cisterna chyli and into the thoracic duct^2,5,7,11,15^. In this study, the patient was a 67-year-old male with previous chronic pancreatitis who underwent pancreatoduodenectomy for ductal adenocarcinoma of the pancreatic head.

The lymphatic system of some organs (soft tissues, liver, and intestine) all communicate with each other and occasionally drain into the cisterna chyli and the thoracic duct. Intraperitoneal chylous leakage may be associated with painless abdominal distension, significant fluid loss, protein loss, severe malnutrition, and susceptibility to infection. Paracentesis can confirm chylous ascites if the ascitic fluid is milky, sterile, and with increased levels of triglycerides and protein^4,8,13^.

Prevention of chylous ascites following pancreatoduodenectomy with lymphadenectomy for pancreatic adenocarcinoma is possible if after removal of the specimen by careful evaluation of any lymphatic leak in the operative field, intraoperatively. During resection, all lymphatic vessels should be identified and clipped or ligated before cutting^2,11^.

Despite the clear understanding of chylous ascites, no specific guidelines are available for the management of chylous ascites due to the rarity of the condition and the paucity of cases^8,11^. The initial treatment is frequently conservative, which includes dietary modifications, TPN, use of somatostatin analogs, and paracentesis. Dietary management includes medium-chain triglycerides and high-protein and low-fat intake. These classes of triglycerides are directly absorbed into the portal circulation, lowering the flow in the intestinal lymphatic system. The main indication of TPN is when the patient does not tolerate an oral diet. In some cases, early TPN has led to a faster resolution^3,9,12^.

One of the indications of interventional radiology and surgical intervention is when conservative treatments fail. Patients with high drain output (>1 L/day on presentation) and patients with persistent chylous ascites despite conservative treatment. The technique of intranodal lymphangiography is performed by ultrasonography accessing the inguinal lymph nodes and injection of lipiodol contrast to identify the exact source of the chylous leak^6,12,15^.

As soon as the leak is identified, it can be embolized with a combination of coils and glue at the level of the node or the lymphatic vessel if technically feasible. Lipiodol lymphangiography and embolization were performed in this case and the postoperative course was uneventful. In surgical intervention, the leak is identified and should be clipped or ligated^2,11^.

## CONCLUSION

Chylous ascites is a rare and important complication following pancreatoduodenectomy. Lipiodol lymphangiography and embolization should be performed in patients who are not responding to conservative measures.
